# Supplementary material for: Terahertz Spectroscopy: An Investigation of the Structural Dynamics of Freeze-Dried Poly Lactic-co-glycolic Acid Microspheres
Source: Pharmaceutics. 2019 Jun 20;11(6):291. doi: 10.3390/pharmaceutics11060291 (PMC6631728; doi:10.3390/pharmaceutics11060291)
Supplement: Supplementary file 1 [file pharmaceutics-11-00291-s001.pdf]

Supplementary Information to Terahertz Spectroscopy: An investigation of the Structural Dynamics of Freeze-Dried Poly Lactic-co-glycolic Acid Microspheres

Talia A. Shmool,† Philippa J. Hooper,† Gabriele S. Kaminski Schierle,†
Christopher F. van der Walle,‡ and J. Axel Zeitler*,¶

†*Department of Chemical Engineering and Biotechnology, University of Cambridge, Philippa Fawcett Drive, Cambridge, CB3 0AS, United Kingdom*

‡*Biopharmaceutical Development, AstraZeneca, Granta Park, Cambridge, CB21 6GH, United Kingdom*

¶*Department of Chemical Engineering and Biotechnology, University of Cambridge, Philippa Fawcett Drive, Cambridge CB3 0AS, United Kingdom*

E-mail: jaz22@cam.ac.uk

Phone: +44 1223 334783

Scanning Electron Microscopy Characterization

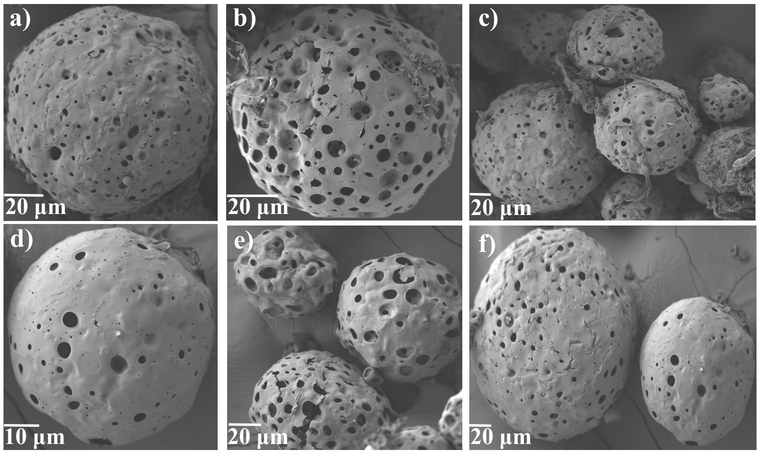

Figure S1: Representative SEM micrographs for blank PLGA 50:50 microspheres shown in a), b), and c) and blank PLGA 75:25 microspheres shown in d), e) and f).

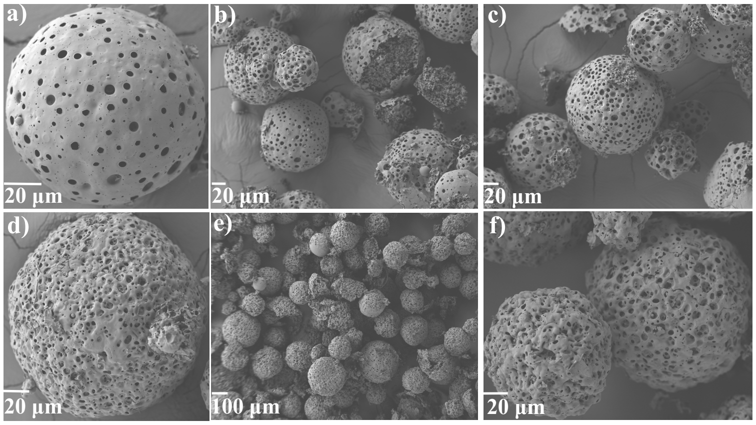

Figure S2: Representative SEM micrographs for low exendin-4 loaded PLGA 50:50 microspheres shown in a), b), and c) and high exendin-4 loaded PLGA 50:50 microspheres shown in d), e) and f).

Fourier Transform Infrared Spectroscopy

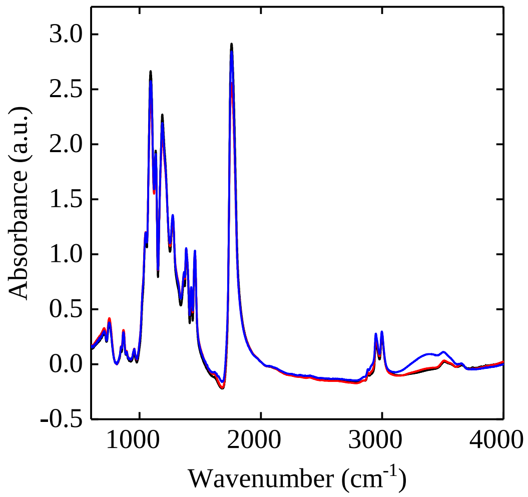

Figure S3: FTIR spectra of blank microspheres (solid black line), low polypeptide loaded (solid red line) and high polypeptide loaded (solid blue line) PLGA 75:25 microspheres.

MDSC Data

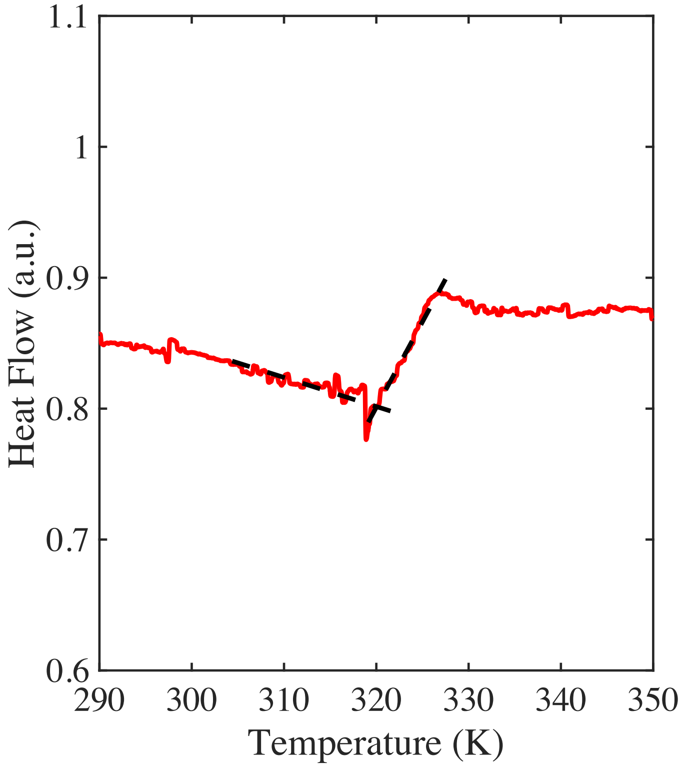

Figure S4: MDSC thermogram of PLGA 50:50 blank microsphere.

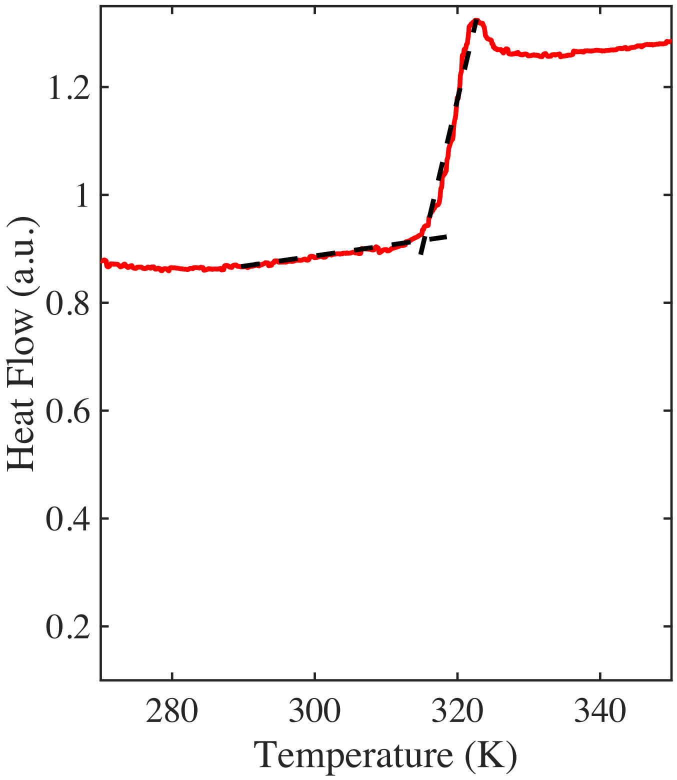

Figure S5: MDSC thermogram of PLGA 50:50 low peptide loading microsphere.

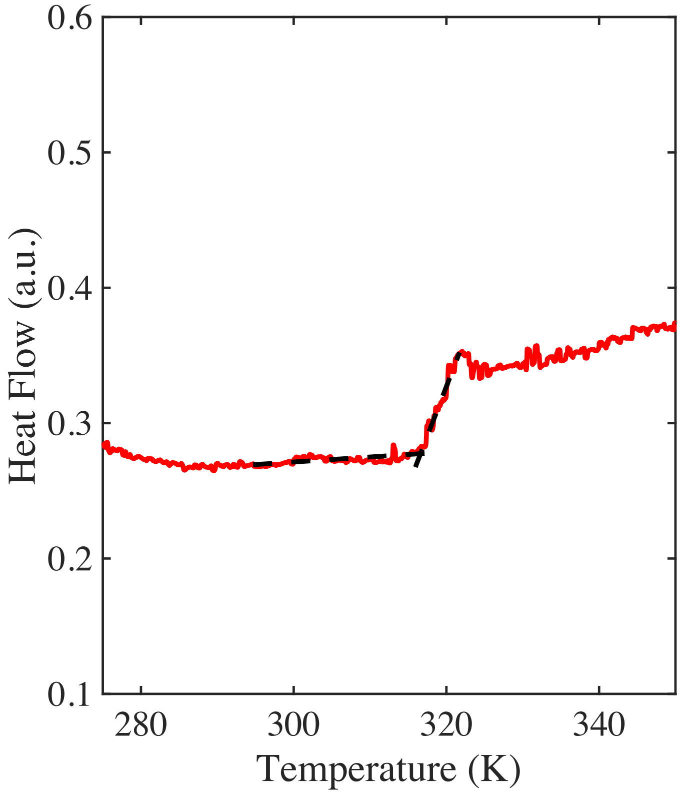

Figure S6: MDSC thermogram of PLGA 50:50 high peptide loading microsphere.

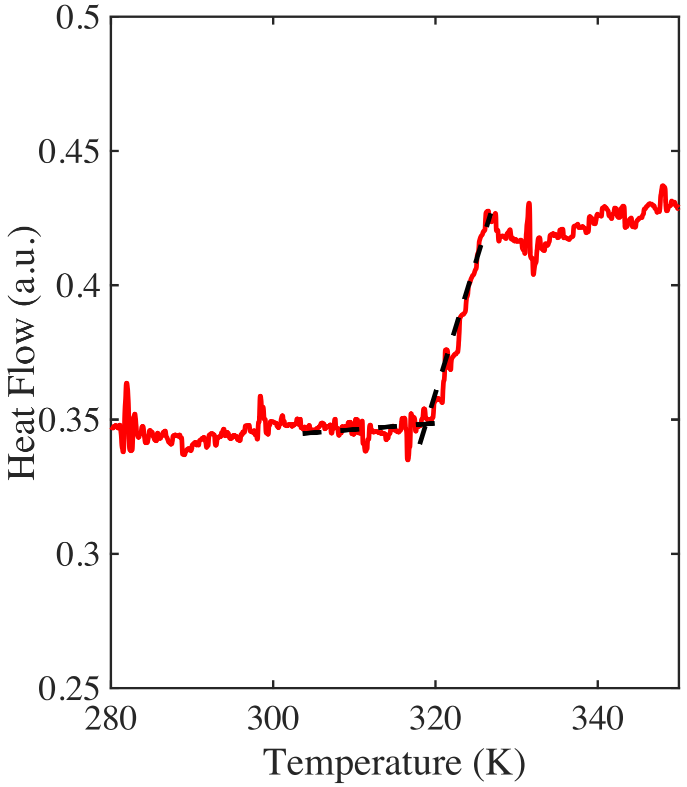

Figure S7: MDSC thermogram of PLGA 75:25 blank microsphere.

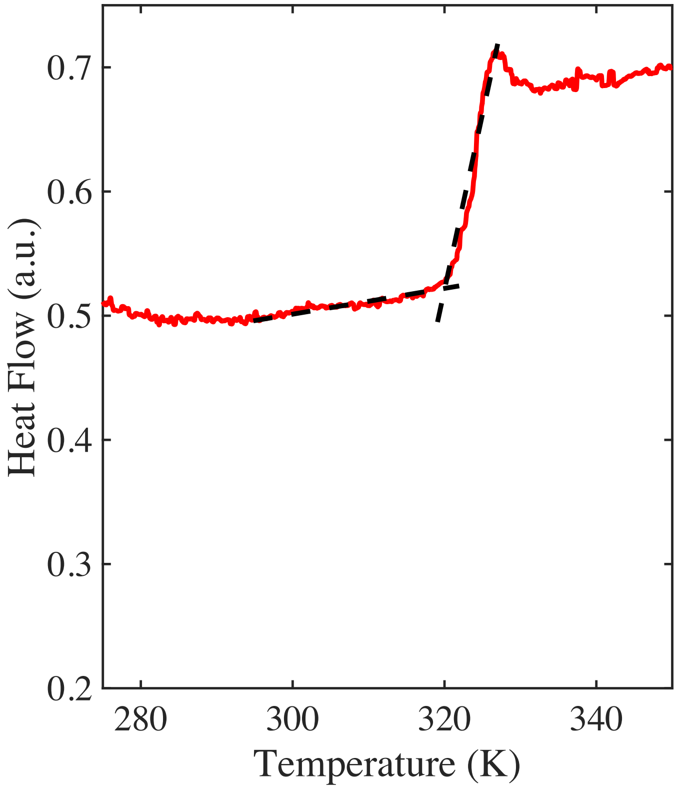

Figure S8: MDSC thermogram of PLGA 75:25 low peptide loading microsphere.

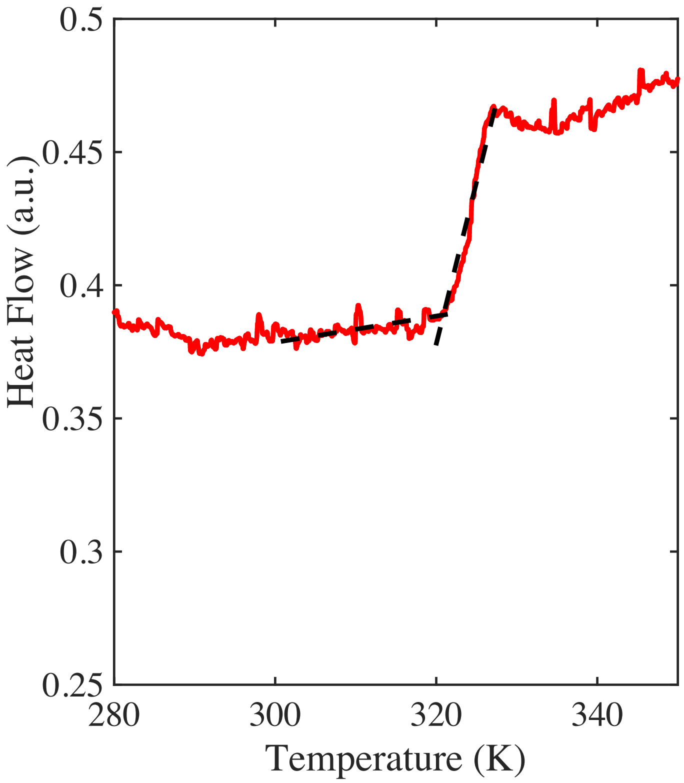

Figure S9: MDSC thermogram of PLGA 75:25 high peptide loading microsphere.

Terahertz Spectroscopy Data

Absorption coefficient and refractive index spectra of all materials between $0.3 - 3\,\text{THz}$, in the temperature range of $90 - 370\,\text{K}$, with $10\,\text{K}$ temperature increments between spectra. The sample thickness was in the range of $300 - 600$ micrometers. The absorption coefficient spectra include the upper limit of detectable absorption which is indicated by the black dashed line. Both the absorption spectra and the refractive index spectra are ordered from highest to lowest temperatures, with red lines indicating the high temperatures and blue lines indicating the low temperatures.

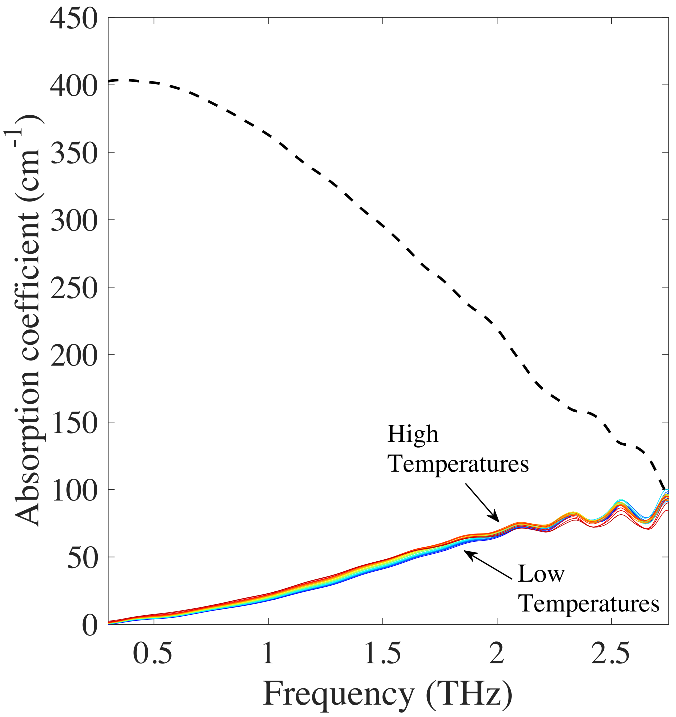

Figure S10: Terahertz absorption spectra of a blank PLGA 75:25 microsphere sample over $0.3 - 2.8$ THz in the temperature range of $100 - 360$ K.

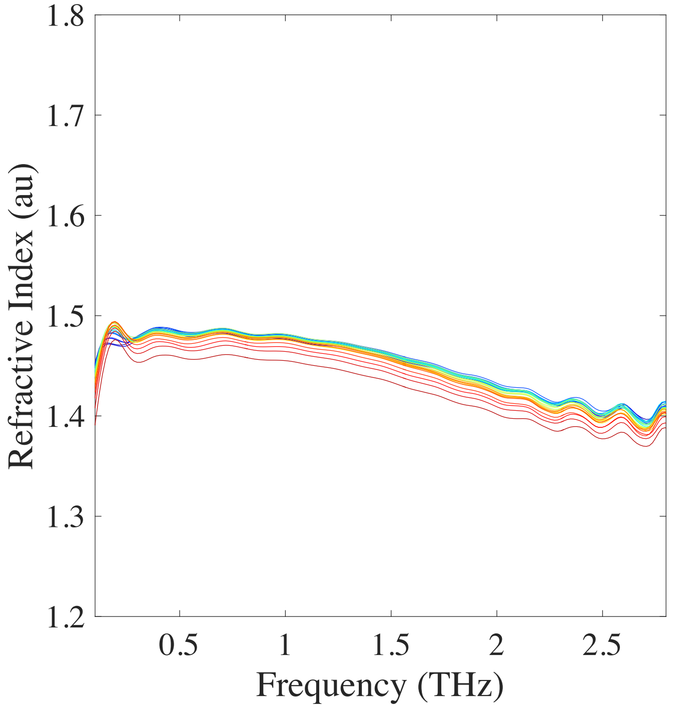

Figure S11: Refractive index spectra of a blank PLGA 75:25 microsphere sample over $0.1 - 2.8$ THz in the temperature range of $100 - 360$ K.

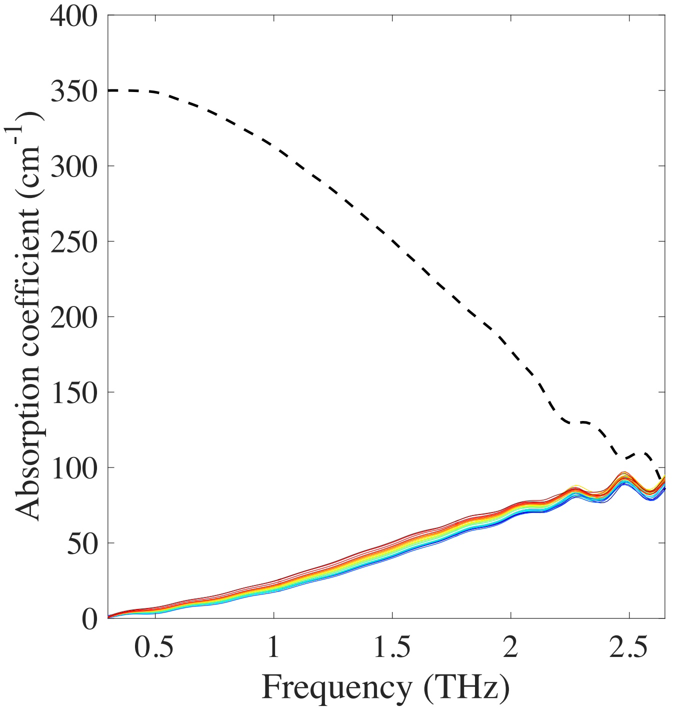

Figure S12: Terahertz absorption spectra of a blank PLGA 50:50 microsphere sample over $0.3-2.8\,\mathrm{THz}$ in the temperature range of $100-350\,\mathrm{K}$.

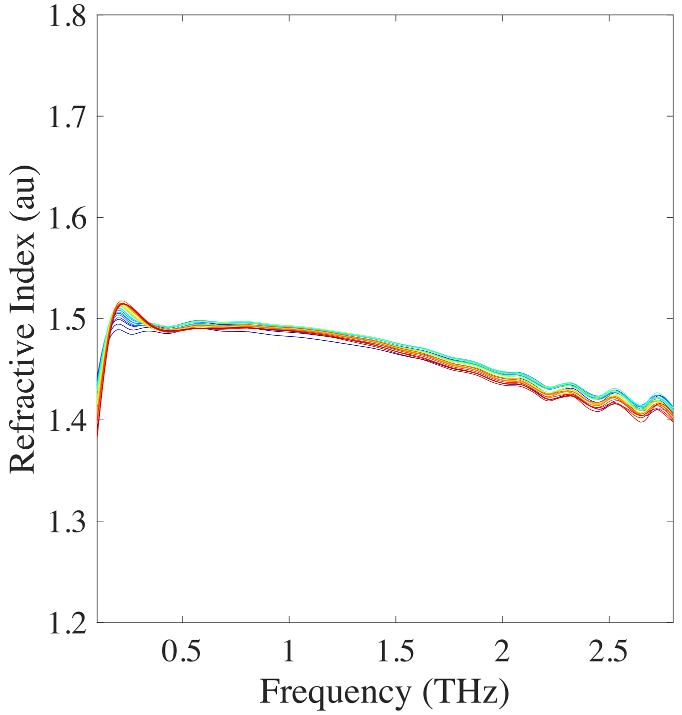

Figure S13: Refractive index spectra of a blank PLGA 50:50 microsphere sample over $0.1-2.8\,\mathrm{THz}$ in the temperature range of $100-350\,\mathrm{K}$.

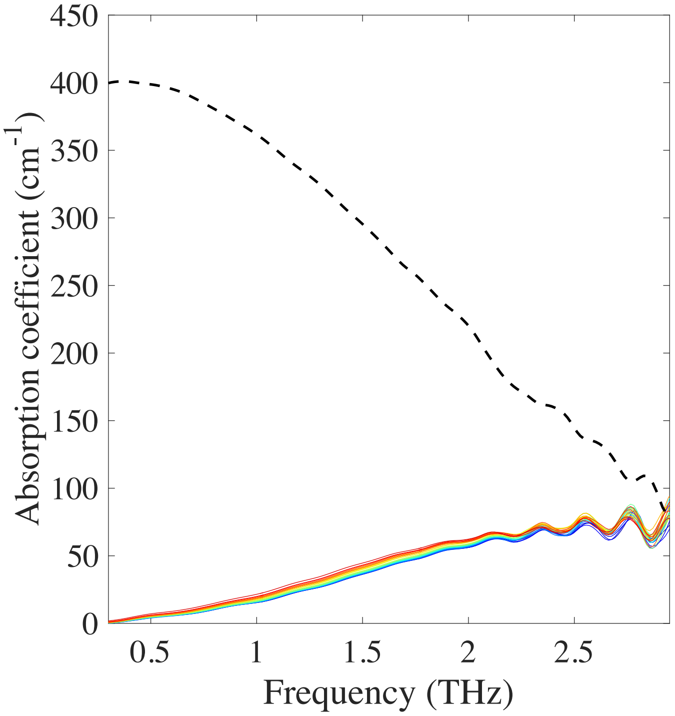

Figure S14: Terahertz absorption spectra of a low polypeptide loaded PLGA 75:25 microsphere sample over $0.3 - 2.8\,\mathrm{THz}$ in the temperature range of $100 - 350\,\mathrm{K}$.

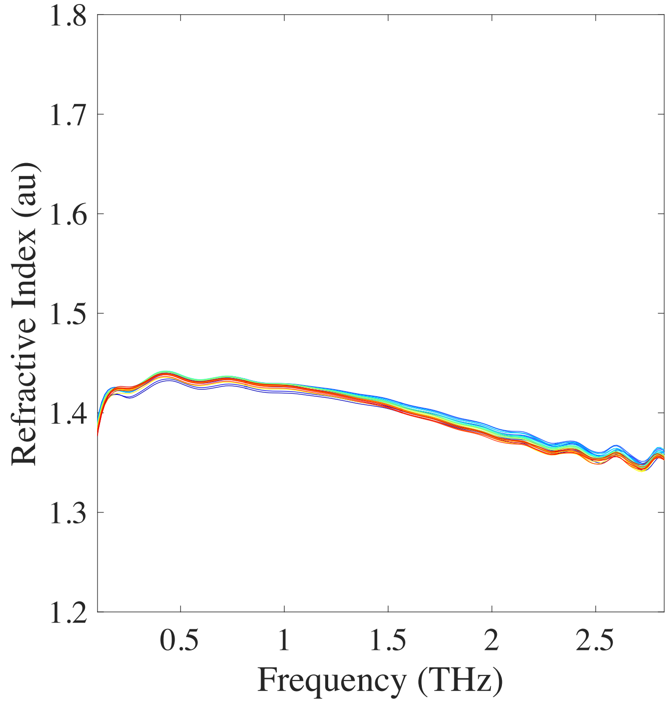

Figure S15: Refractive index spectra of a low polypeptide loaded PLGA 75:25 microsphere sample over $0.1 - 2.8\,\mathrm{THz}$ in the temperature range of $100 - 350\,\mathrm{K}$.

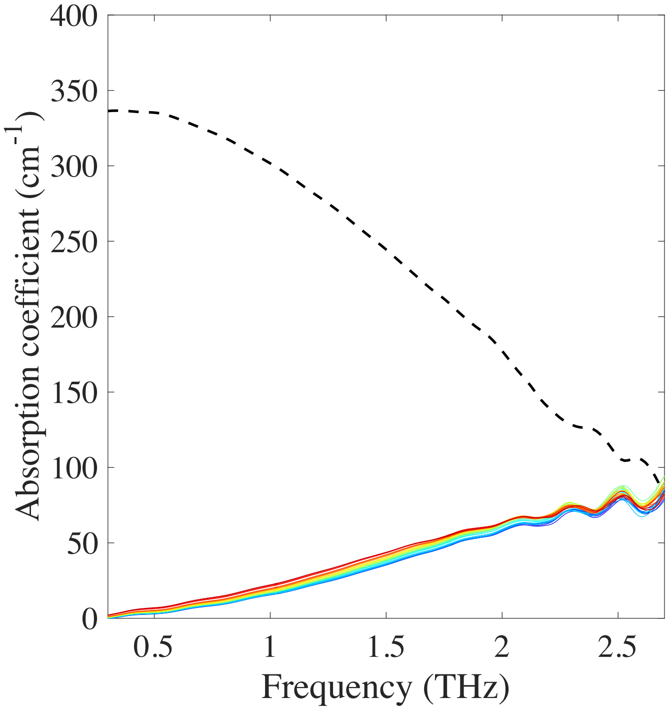

Figure S16: Terahertz absorption spectra of a low polypeptide loaded PLGA 50:50 microsphere sample over $0.3 - 2.8\,\mathrm{THz}$ in the temperature range of $100 - 370\,\mathrm{K}$.

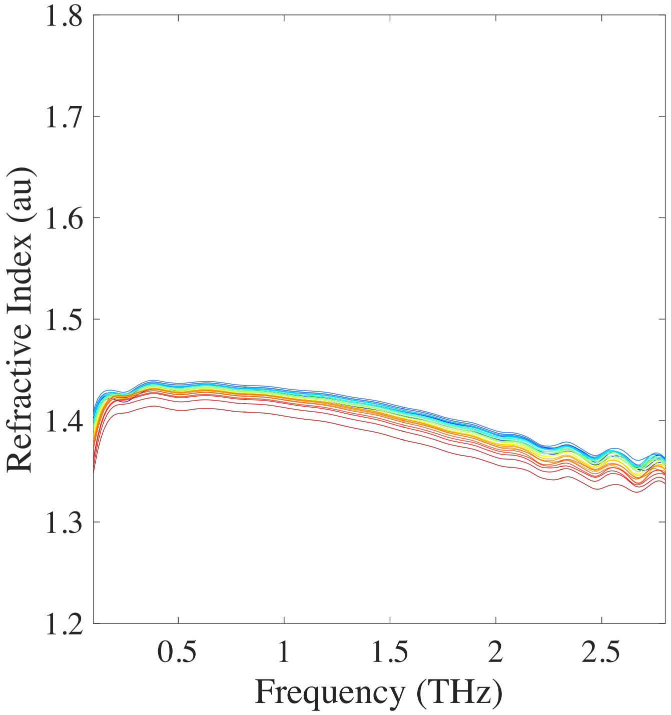

Figure S17: Refractive index spectra of a low polypeptide loaded PLGA 50:50 microsphere sample over $0.1 - 2.8\,\mathrm{THz}$ in the temperature range of $100 - 350\,\mathrm{K}$.

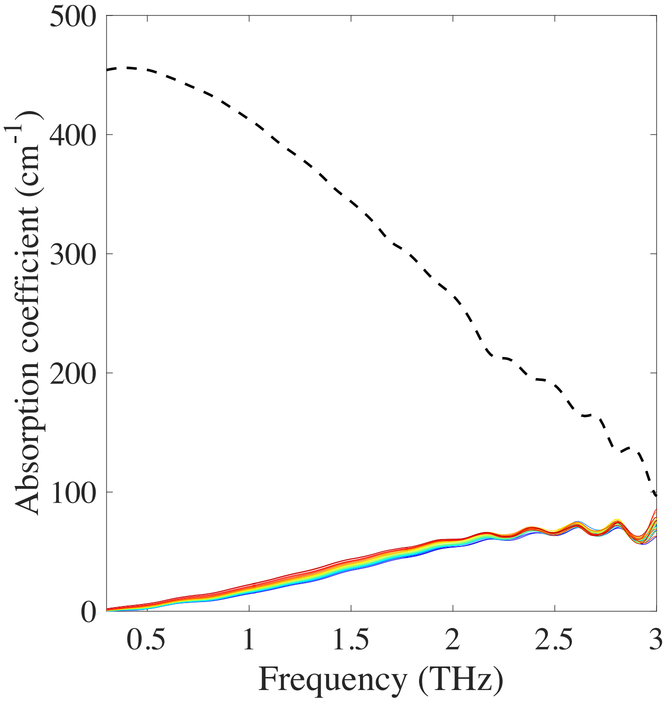

Figure S18: Terahertz absorption spectra of a low polypeptide loaded PLGA 75:25 microsphere sample over $0.3 - 370\,\mathrm{THz}$ in the temperature range of $100 - 350\,\mathrm{K}$.

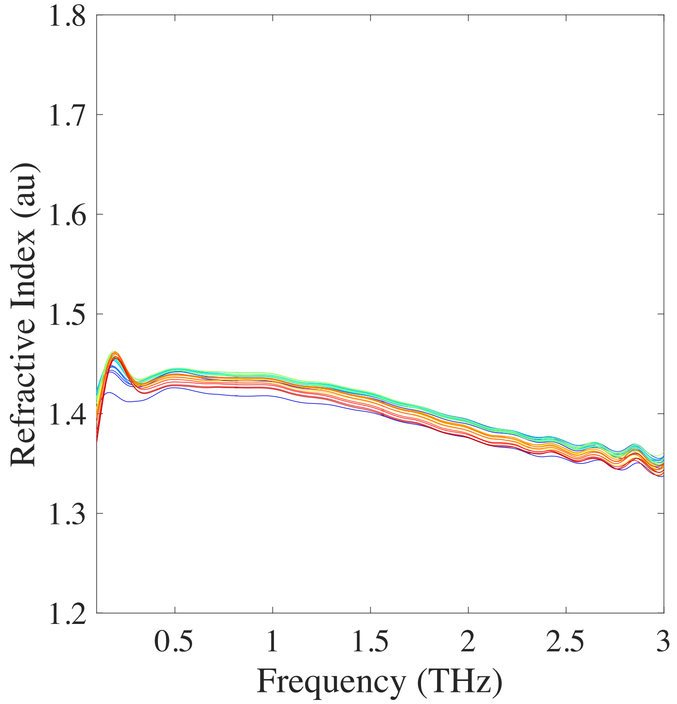

Figure S19: Refractive index spectra of a low polypeptide loaded PLGA 75:25 microsphere sample over $0.1 - 3\,\mathrm{THz}$ in the temperature range of $100 - 370\,\mathrm{K}$.

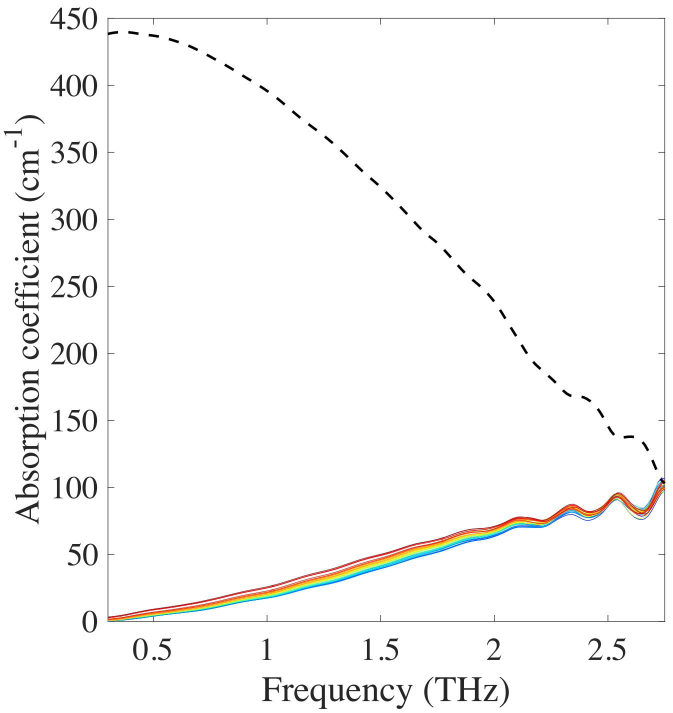

Figure S20: Terahertz absorption spectra of a high polypeptide loaded PLGA 50:50 microsphere sample over $0.3 - 2.8\,\mathrm{THz}$ in the temperature range of $100 - 370\,\mathrm{K}$.

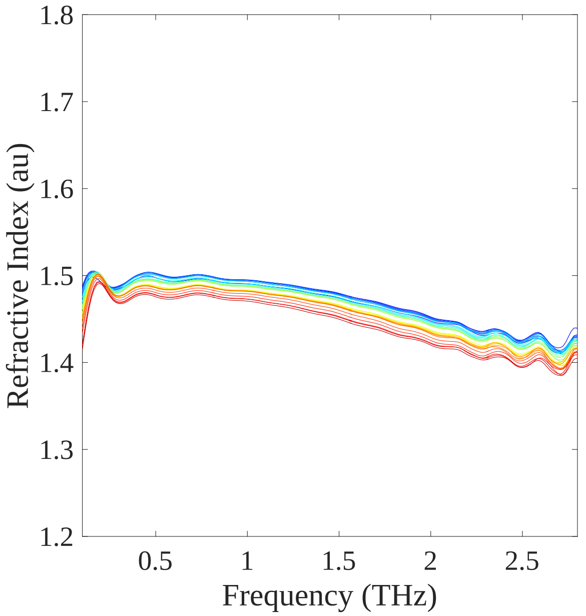

Figure S21: Refractive index spectra of a high polypeptide loaded PLGA 50:50 microsphere sample over $0.1 - 2.8\,\mathrm{THz}$ in the temperature range of $100 - 370\,\mathrm{K}$.

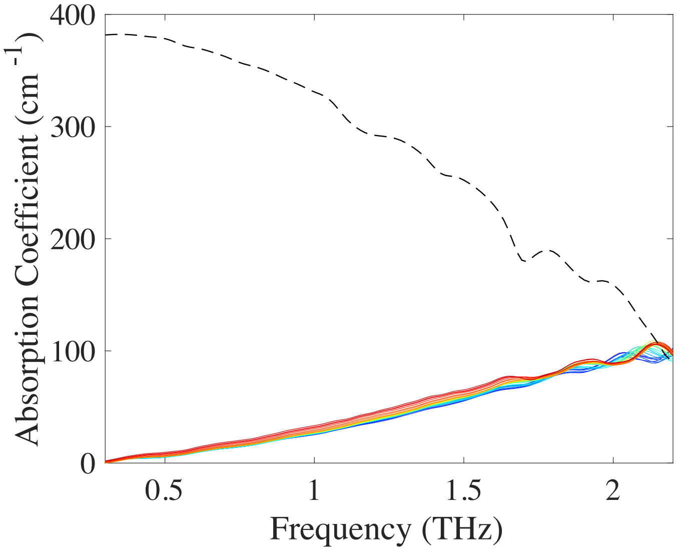

Figure S22: Terahertz absorption spectra of a medium MW PLGA 50:50 over $0.3 - 2.2\,\mathrm{THz}$ in the temperature range of $90 - 360\,\mathrm{K}$.

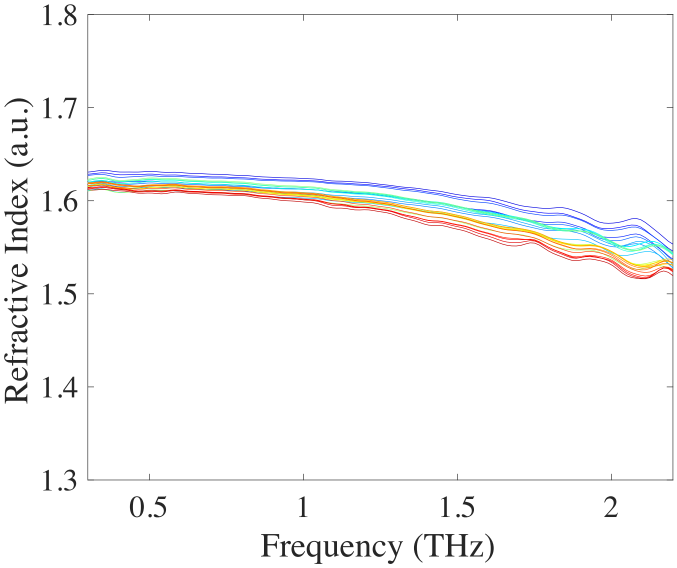

Figure S23: Refractive index spectra of a medium MW PLGA 50:50 over $0.3 - 2.2\,\mathrm{THz}$ in the temperature range of $90 - 360\,\mathrm{K}$.

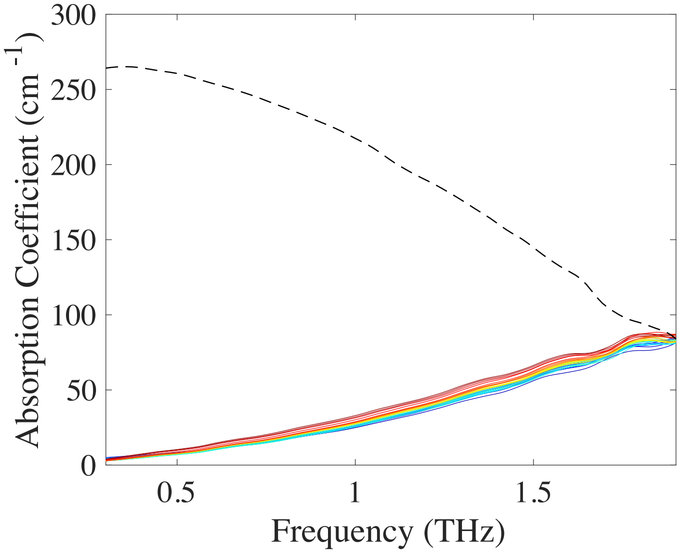

Figure S24: Terahertz absorption spectra of a medium MW PLGA 75:25 sample over $0.3 - 1.9\,\mathrm{THz}$ in the temperature range of $90 - 350\,\mathrm{K}$.

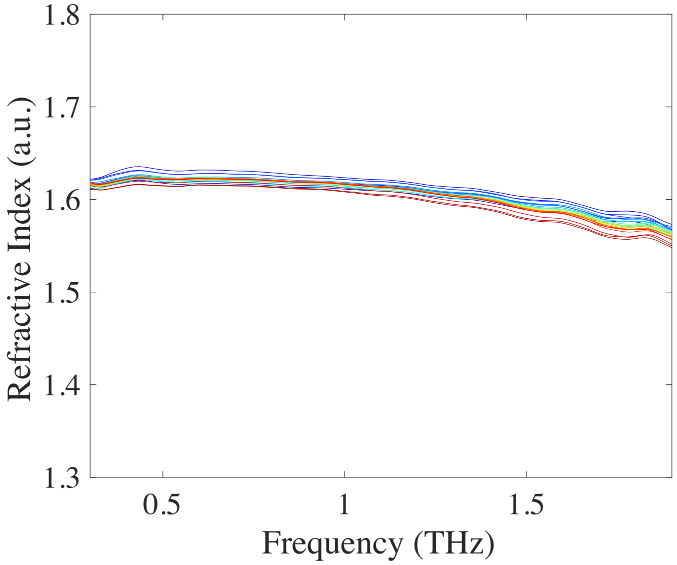

Figure S25: Refractive index spectra of a medium MW PLGA 75:25 sample over 0.3−1.9 THz in the temperature range of $90 - 350\,\mathrm{K}$.
